# Short-form video platforms as a source of ankylosing spondylitis information: a cross-sectional content analysis

**DOI:** 10.3389/fdgth.2026.1757584

**Published:** 2026-03-04

**Authors:** Xi Wei, Zhenfu Sun, Zilin Zhu, Luoxi Zheng, Donghai Zhou, Muzhi Chen

**Affiliations:** 1 The Second Clinical Medical College, Zhejiang Chinese Medical University, Hangzhou, Zhejiang, China; 2 The Fourth Clinical Medical College, Xinjiang Medical University, Urumqi, China; 3 Department of Rheumatology, The Second Affiliated Hospital of Zhejiang Chinese Medical University, Hangzhou, Zhejiang, China

**Keywords:** ankylosing spondylitis, China, health communication, short-video, social media, video quality

## Abstract

**Background:**

Short-video platforms have become major channels for health information dissemination, yet the quality and reliability of content on ankylosing spondylitis (AS) remain underexplored.

**Objective:**

This study aimed to systematically evaluate the quality, reliability, and characteristics of AS-related short videos on three major Chinese platforms: TikTok, Bilibili, and rednote.

**Methods:**

A cross-sectional content analysis was conducted on 300 videos (100 per platform) collected in November 2025. Video uploaders were categorized as professional physicians, non-professional physicians, individual users, or institutions. Four validated instruments—modified DISCERN (mDISCERN), Global Quality Scale (GQS), Journal of the American Medical Association (JAMA) criteria, and Video Information Quality Index (VIQI)—were used to assess reliability, completeness, and production quality. User engagement metrics, including likes, shares, comments, and collections, were also analyzed.

**Results:**

Most videos were uploaded by professional physicians and primarily focused on clinical manifestations and treatment. Videos presented in animated or lecture-style formats demonstrated higher information quality and reliability, whereas casually recorded videos consistently scored lower across multiple assessment tools. Overall, the quality of AS-related short videos was moderate. In unadjusted analyses, collections were positively associated with information quality, suggesting that users may preferentially retain more informative content. However, after accounting for platform characteristics and exposure-related factors, information quality was not a consistent independent driver of relative engagement, and mDISCERN showed an inverse association with standardized collection levels. Other engagement indicators, including likes, comments, shares, and follower counts, showed weak or inconsistent relationships with video quality.

**Conclusion:**

Although AS-related short videos are predominantly produced by physicians, the overall quality remains suboptimal. Information quality appears to influence certain user behaviors, such as content saving, but does not consistently translate into higher overall engagement. These findings highlight the limitations of using engagement metrics as proxies for content quality. Users should prioritize content from verified medical professionals, and platforms may consider integrating quality-oriented indicators and improving certification systems to enhance health information dissemination.

## 1 Introduction

Ankylosing spondylitis (AS) is a chronic, inflammatory, and autoimmune disease primarily affecting the sacroiliac joints and spine, and it may also involve multiple extra-articular systems such as the eyes, lungs, and gastrointestinal tract (1). In China, the prevalence of AS is approximately 0.29% (2), with a markedly higher incidence in men—approximately 2.8 times that observed in women (3). AS is one of the leading causes of impaired work capacity and reduced quality of life among young and middle-aged adults, particularly men (4). Early manifestations such as inflammatory back pain are often nonspecific and insidious, and therefore easily misdiagnosed as mechanical strain or nonspecific low back pain, resulting in substantial diagnostic delay or even misdiagnosis (5). Without timely and standardized intervention, AS carries a high risk of disability; in advanced stages, patients may develop complete spinal ankylosis and deformity, leading to irreversible functional impairment. Thus, early diagnosis and continuous self-management are of critical importance for controlling inflammation, slowing disease progression, and preventing long-term disability.

In the digital era, the pathways through which the public obtains health information have been fundamentally reshaped. The internet and social media have become major sources of knowledge for patients seeking to understand and manage their conditions (6, 7). Short-video platforms, in particular, have emerged as influential health communication channels due to their enormous user bases, visually engaging formats, and algorithm-driven dissemination mechanisms (8). This trend is especially relevant for AS, which predominantly affects young and middle-aged males—the most active demographic on short-video platforms (9). When experiencing unexplained chronic back pain, individuals in this group often turn first to platforms such as TikTok (Chinese version), Bilibili, or rednote for information. However, despite their convenience, these platforms pose substantial risks: the quality of health content is highly variable, and partial, inaccurate (10–12), or commercially biased information may be widely propagated, potentially influencing patients' health-seeking behavior, treatment adherence, and overall health outcomes (13–16). Although previous studies have evaluated similar issues in social media content related to other rheumatic diseases (17, 18), AS-related content on Chinese short-video platforms remains insufficiently explored.

To address this gap, the present study conducted a cross-sectional content analysis to systematically evaluate and compare the quality and reliability of AS-related short videos on three of China's most widely used platforms: TikTok, Bilibili, and rednote. A set of widely adopted and validated assessment instruments was applied to capture multiple dimensions of health information quality and reliability in short-video content. The findings of this study are expected to provide practical guidance for AS patients in identifying trustworthy online information and making more informed health decisions; to support rheumatologists and other healthcare professionals in developing precise and effective digital patient education strategies; and to offer empirical evidence for platform administrators seeking to optimize the health information ecosystem and establish robust quality-control mechanisms.

## 2 Methods

### 2.1 Ethical considerations

This study analyzed only publicly accessible data obtained from short-video platforms and did not involve human participants, interventions, or sensitive personal information. Therefore, formal ethical approval was not required.

### 2.2 Study design

A cross-sectional content analysis was conducted to systematically evaluate the information quality, reliability, and content characteristics of AS-related short videos published on three major Chinese social media platforms: TikTok, Bilibili, and rednote. The official name of TikTok's mainland China version is Douyin, but given that the two platforms operate as region-specific versions of the same product, and to maintain consistency with terminology widely adopted in the global academic literature, the term TikTok is used throughout this manuscript to refer to the data source platform.

### 2.3 Data sources and search strategy

Video data were collected from three major Chinese social media platforms: TikTok, Bilibili, and rednote, between 15 November 2025 and 18 November 2025. To reduce the influence of personalized recommendation algorithms, all searches were conducted using newly registered accounts with no prior browsing history, content interactions, or followed accounts. All searches were conducted by the same investigator on a single device, with the device location set to Hangzhou, China, and the platform language configured to Chinese. No manual cache clearing was performed prior to the searches. A single standardized Chinese disease-specific keyword, “强直性脊柱炎” (ankylosing spondylitis), was used consistently across all platforms. No additional synonyms or alternative keywords were applied, and hashtags were not used. Searches were restricted to the video content section corresponding to the entered keyword, excluding other content formats such as image–text posts or live streams. Search results were reviewed using each platform's default sorting option at the time of data collection, with Bilibili applying comprehensive ranking and TikTok and rednote using their respective default recommendation-based ranking systems. All searches were conducted within a single predefined time window. For each platform, the first 100 consecutively ranked videos returned by the search results were initially screened. Videos were assessed according to predefined inclusion and exclusion criteria. When ineligible videos were identified within the initial top 100 results, additional videos were screened sequentially from lower-ranked positions until a final set of 100 eligible videos per platform was obtained. Duplicate videos were identified and removed during the screening process. Duplicates were operationally defined as videos uploaded by the same account with identical video titles and identical audiovisual content. Following this stepwise screening procedure, a final dataset of 100 unique videos per platform was included, yielding a total sample of 300 videos for analysis.

### 2.4 Inclusion and exclusion criteria

Videos were eligible for inclusion if they met both of the following criteria:

the primary language was Chinese;
the core content focused on AS-related medical knowledge, diagnosis, treatment, disease management, or patient experience.

Videos were excluded if they met any of the following conditions:

duplicate content;
content unrelated to AS;
purely commercial promotional material lacking substantive educational value.

### 2.5 Data extraction

Two trained reviewers independently extracted data using a standardized data collection form, and discrepancies were resolved through discussion with a third reviewer. Variables extracted included:

Basic characteristics: platform, video duration, **video upload time**, engagement metrics (likes, collections, comments, shares), and follower count of the video uploader.

Uploader classification: uploader identity was determined based on a combination of platform verification badges, self-described professional information in user profiles, and the content of the videos. Professional physicians were defined as uploaders who were verified by the platform or clearly identified themselves as rheumatologists or orthopedists with identifiable professional credentials. Non-professional physicians included other healthcare providers, such as general practitioners, nurses, or rehabilitation therapists, who disclosed a healthcare background but were not specialists in rheumatology or orthopedics. Institution accounts were defined as official accounts representing hospitals, companies, academic societies, or patient organizations. Uploaders without verifiable medical credentials or institutional affiliation were classified as individual users, including patients, caregivers, or non-medical content creators. No separate “unknown” category was used; ambiguous cases were conservatively classified as individual users.

Video presentation form: highlight presentation, interview, video recording, scene reenactment, animated video, speech, news, and documentary film.

Content completeness: each video was evaluated for coverage of seven core medical domains, including epidemiology, etiology, clinical manifestations, diagnosis, treatment, prevention, and prognosis.

### 2.6 Quality and reliability assessment

Four internationally recognized instruments were used to evaluate the quality and reliability of each video: the Global Quality Score (GQS), the modified DISCERN (mDISCERN), the Video Information Quality Index (VIQI), and the Journal of the American Medical Association (JAMA) benchmark criteria. The detailed scoring criteria for each instrument are provided in Supplementary Tables S1–S4. The GQS is a global 5-point Likert-type scale designed to assess the overall educational value, information quality, and presentation flow of health-related content, with higher scores indicating better overall quality. The total GQS score for each video ranges from 1 to 5 (19, 20). The mDISCERN was applied as a simplified and context-adapted version of the original DISCERN instrument to assess the reliability of health information in short-form videos. The modified tool consists of five equally weighted items, each scored dichotomously (yes = 1, no = 0), yielding a total score ranging from 0 to 5, and has been widely used in prior studies evaluating the reliability of online video-based health information (21, 22). Given the brief, fragmented, and non-textual nature of short videos, many video-based evaluations have adopted simplified instruments rather than applying the full-length DISCERN directly, and mDISCERN has also been used in Chinese-language short-video platform studies (23). The JAMA benchmark criteria were used to assess information credibility based on four items—authorship, attribution, currency, and disclosure—each scored as present (1) or absent (0), yielding a total score ranging from 0 to 4 (24, 25). The Video Information Quality Index (VIQI) was applied to evaluate video-specific educational quality across four domains (information flow, information accuracy, technical quality, and goal precision), with total scores ranging from 4 to 20, where higher scores indicate better quality (26).

All reviewers underwent calibration and pilot testing prior to formal scoring to ensure consistency. Inter-rater reliability between the two independent reviewers was formally assessed. For the four quality assessment instruments (GQS, mDISCERN, JAMA, and VIQI), intraclass correlation coefficients (ICCs) were calculated based on the total scores assigned by each reviewer using a two-way random-effects model with absolute agreement for single measurements. For content completeness, which was evaluated as the presence or absence of information across seven core medical domains (epidemiology, etiology, clinical manifestations, diagnosis, treatment, prevention, and prognosis), inter-rater agreement was assessed using Cohen's kappa coefficients. All inter-rater reliability analyses were conducted using the original independent ratings provided by the two reviewers, prior to discrepancy resolution by the third reviewer. Inter-rater reliability results for the quality assessment instruments are presented in Table 1, and agreement for content completeness domains is provided in Supplementary Table S5.

**Table 1:** Inter-rater reliability of video quality assessment instruments.

Instrument	ICC (95% CI)
GQS	0.735 (0.678–0.783)
mDISCERN	0.900 (0.876–0.919)
JAMA	0.818 (0.776–0.852)
Viqi	0.977 (0.971–0.981)

Intraclass correlation coefficients (ICCs) were calculated using a two-way random-effects model with absolute agreement for single measurements.

### 2.7 Statistical analysis

Continuous variables were summarized as mean ± standard deviation or median (interquartile range) depending on distribution. Categorical variables were reported as frequencies and percentages. Between-group comparisons were conducted using independent-samples t tests, Mann–Whitney U tests, Kruskal–Wallis H tests with Dunn's *post-hoc* tests, or chi-square/Fisher's exact tests as appropriate. Spearman's rank correlation was used to examine associations between video quality scores and user engagement metrics. A two-tailed *P* < 0.05 was considered statistically significant. All analyses were performed using R software (version 4.3.2). Given the substantial structural and algorithmic differences across platforms, additional analyses were conducted to improve the interpretability of engagement comparisons. To address the limited comparability of raw engagement metrics, collection counts were log-transformed [log(x ± 1)] and standardized within each platform by conversion to z-scores, reflecting relative engagement levels. Cross-platform differences in standardized engagement were assessed using the Kruskal–Wallis test. Video upload time was operationalized as days since upload, defined as the number of days between the video upload date and the end of the data collection period. To examine whether information quality was independently associated with user engagement after accounting for potential confounding factors, a multivariable linear regression analysis was performed. The platform-wise standardized collection score (z_log_collections) was used as the dependent variable. The mDISCERN score was included as the primary independent variable, while platform (categorical), days since upload, follower count of the video uploader (log-transformed), and video duration were entered as covariates.

## 3 Results

### 3.1 General characteristics of the videos

**Figure 1:**
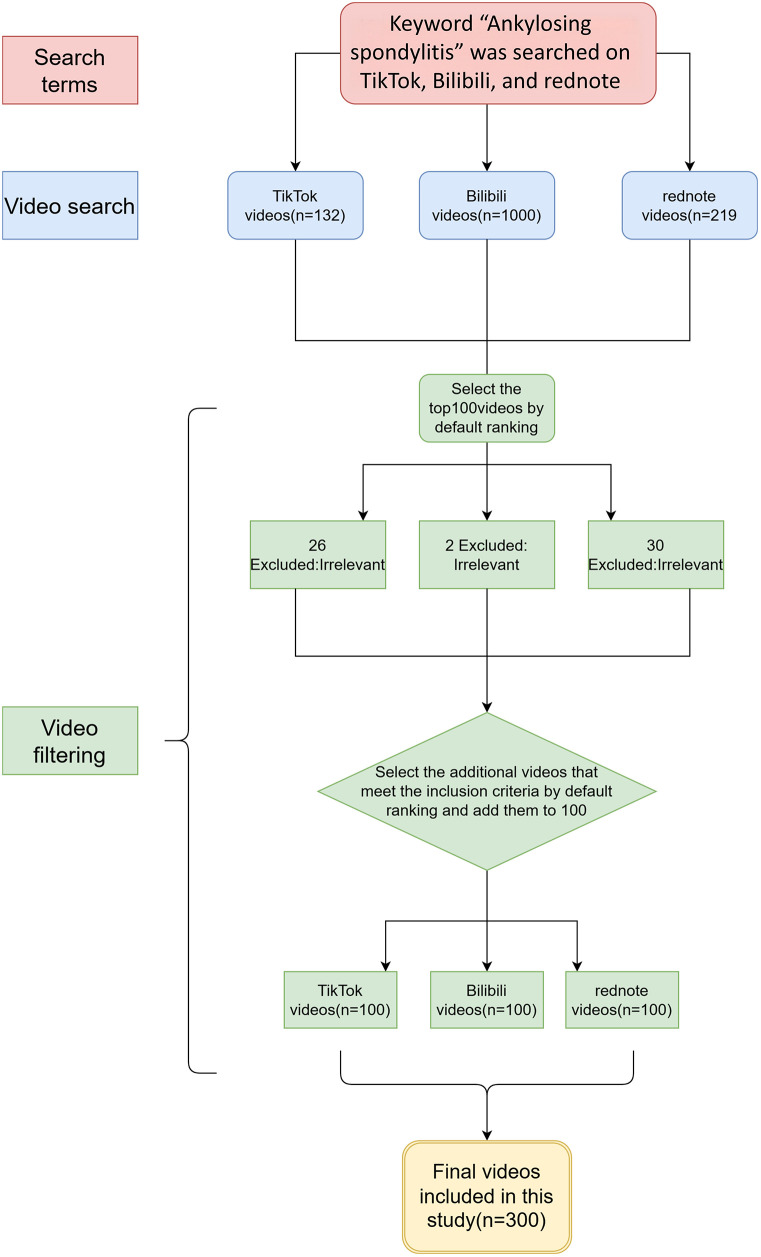
Flowchart of the study.

As shown in Figure 1, searches for “ankylosing spondylitis” identified 132 videos on TikTok, 1,000 on Bilibili, and 219 on rednote. After removing advertisements, duplicates, and irrelevant content, 100 videos were included from each platform, yielding a total of 300 AS-related videos for analysis. TikTok videos received significantly higher numbers of likes, comments, shares, collections, and higher follower counts of video uploaders compared with those on Bilibili and rednote (all *P* < .001). In contrast, Duration was significantly longer on Bilibili than on TikTok or rednote (*P* < .001). The mDISCERN score was higher for Bilibili videos than for those on TikTok and rednote (mean 2.62, SD 0.86 vs. mean 2.10, SD 0.99 vs. mean 2.49, SD 0.82; *P* < .001). No significant differences were found among platforms in GQS, JAMA, or VIQI scores. After platform-wise standardization of collection counts, no significant difference in relative collection levels was observed across TikTok, Bilibili, and rednote (Kruskal–Wallis *χ*^2^ = 0.65, *P* = .724), suggesting that the large differences observed in raw engagement metrics were primarily attributable to platform-specific mechanisms. Detailed characteristics across platforms are presented in Table 2.

**Table 2:** General characteristics, unadjusted engagement metrics, and quality scores of ankylosing spondylitis–related videos across platforms.

Parameters	Total (*N* = 300)	TikTok (*n* = 100)	Bilibili (*n* = 100)	Rednote (*n* = 100)	*P*
Duration (seconds), median (IQR)	92.00 (50.75, 190.75)	62.50 (44.75,98.00)	202.50 (128.75,537.00)	70.00 (33.75,98.50)	<.001
Likes, median (IQR)	281.50 (23.00, 2,076.50)	3,077.00 (1,332.25, 10,330.50)	96.00 (24.50,478.25)	22.50 (4.00,226.00)	<.001
Collections, median (IQR)	79.00 (12.75, 701.00)	994.50 (299.50, 2,384.50)	51.00 (15.75,174.00)	10.50 (1.00,70.25)	<.001
Comments, median (IQR)	32.00 (3.00, 295.00)	379.00 (130.75, 1,134.00)	23.00 (4.00,92.50)	3.00 (0.00,11.00)	<.001
Shares, median (IQR)	55.50 (4.00, 724.75)	1,353.50 (357.75, 4,274.00)	23.00 (4.00,89.75)	5.00 (1.00,55.25)	<.001
Follower counts of video uploaders, median (IQR)	24,500.00 (2,912.50, 113,000.00)	90,000.00 (28,500.00, 1,077,000.00)	22,000.00 (630.75, 47,750.00)	8,349.50 (1,126.75, 19,000.00)	<.001
GQS^a^ score, mean (SD)	2.50 ± 0.97	2.51 ± 0.82	2.46 ± 0.86	2.54 ± 1.19	0.841
mDISCERN score, mean (SD)	2.40 ± 0.92	2.10 ± 0.99	2.62 ± 0.86	2.49 ± 0.82	<.001
VIQI^b^ score, mean (SD)	12.09 ± 2.18	11.88 ± 2.40	12.31 ± 2.12	12.08 ± 1.98	0.377
JAMA^c^ score, mean (SD)	2.25 ± 0.65	2.42 ± 0.64	2.19 ± 0.53	2.15 ± 0.74	0.006

a GQS, global quality scale.

b VIQI, video quality instrument.

c JAMA, journal of american medical association.

### 3.2 Video sources and content

As shown in Table 3, uploaders were categorized into Institutions, individual users, professional physicians, and non-professional physicians. Physicians were the predominant uploader group (201/300, 66.9%), with professional physicians contributing more videos (158/300, 52.6%). Among them, orthopedists uploaded more videos (109/158, 69.0%) than rheumatologists (49/158, 31.0%).

**Table 3:** The sources and content of the ankylosing spondylitis–related videos.

Variables	Total (*N* = 300), *n* (%)	TikTok (*n* = 100), *n* (%)	Bilibili (*n* = 100), *n* (%)	Rednote (*n* = 100), *n* (%)	*P*
Video source					<0.001
Individual users	57 (19.0)	10 (17.5)	28 (49.1)	19 (33.3)	
Institutions	42 (14.0)	16 (38.0)	11 (26.1)	15 (35.7)	
Non-Professional physicians	43 (14.3)	21 (48.8)	11 (25.5)	11 (25.5)	
Professional physicians	158 (52.6)	53 (33.5)	50 (31.6)	55 (34.8)	
Different medical specialties					<0.001
Rheumatologist	49 (31.0)	13 (26.5)	9 (18.3)	27 (55.1)	
Orthopedist	109 (69.0)	40 (36.6)	41 (37.6)	28 (25.6)	
Disease knowledge involved	Video Coverage (%, *n* = 300)				-
Epidemiology	14.3% (43/300)	20 (46.5)	14 (32.5)	9 (20.9)	
Etiology	15.0% (45/300)	22 (48.8)	10 (22.2)	13 (28.8)	
Clinical manifestation	66.7% (200/300)	69 (34.5)	62 (31.0)	69 (34.5)	
Diagnosis	26.7% (80/300)	24 (30.0)	30 (37.5)	26 (32.5)	
Treatment	47.7% (143/300)	48 (33.5)	58 (40.5)	37 (25.8)	
Prevention	8.7% (26/300)	23 (88.4)	0 (0.0)	3 (11.5)	
Prognosis	11.0% (33/300)	20 (60.6)	6 (18.1)	6 (21.2)	
Video presentation form					<0.001
Highlight presentation	126 (42.0)	44 (34.9)	40 (31.7)	42 (33.3)	
Interview	20 (6.6)	17 (85.0)	2 (10.0)	1 (5.0)	
Video recording	18 (6.0)	2 (11.1)	7 (38.8)	9 (50.0)	
Scene reenactment	69 (23.0)	19 (27.5)	27 (39.1)	23 (33.3)	
Animated video	24 (8.0)	8 (33.3)	9 (37.5)	7 (29.1)	
Speech	6 (2.0)	1 (16.6)	5 (83.3)	0 (0.0)	
News	23 (7.6)	9 (39.1)	4(17.3)	10(43.4)	
Documentary film	14(4.6)	0(0.0)	6(42.8)	8(57.1)	

Video coverage was calculated as the number of videos addressing the knowledge item divided by the total number of videos (300); the parentheses show the same numerator/300.

Percentages were calculated among professional physicians only.

- Not available.

Regarding content, medical information most commonly addressed clinical manifestations (200/300, 66.7%), followed by treatment (143/300, 47.7%) and diagnosis (80/300, 26.7%). With respect to video presentation form, highlight presentation (126/300, 42.0%) and scene reenactment (23/300, 7.6%) were the most frequently used formats.

### 3.3 Video quality and engagement metrics

#### 3.3.1 Quality assessment

**Figure 2:**
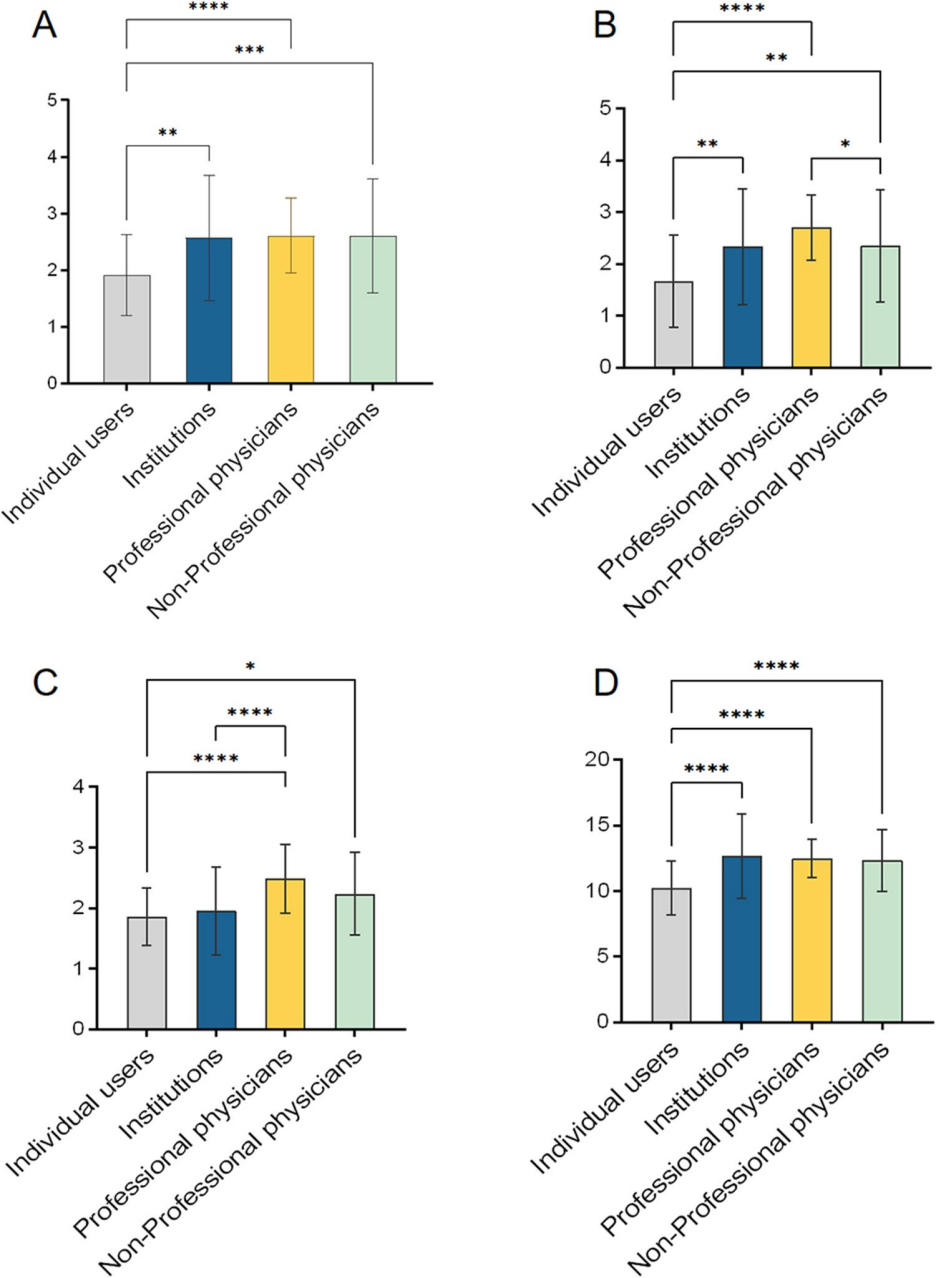
The global quality scale (GQS) score, modified DISCERN score, journal of American medical association (JAMA) score, and video information quality Index (VIQI) score on ankylosing spondylitis from different sources. **(A)** the GQS score, **(B)** the modified DISCERN score, **(C)** the JAMA score, **(D)** the VIQI score, *P < .05, **P < .01, ***P < .001, ****P < 0.0001.

**Figure 3:**
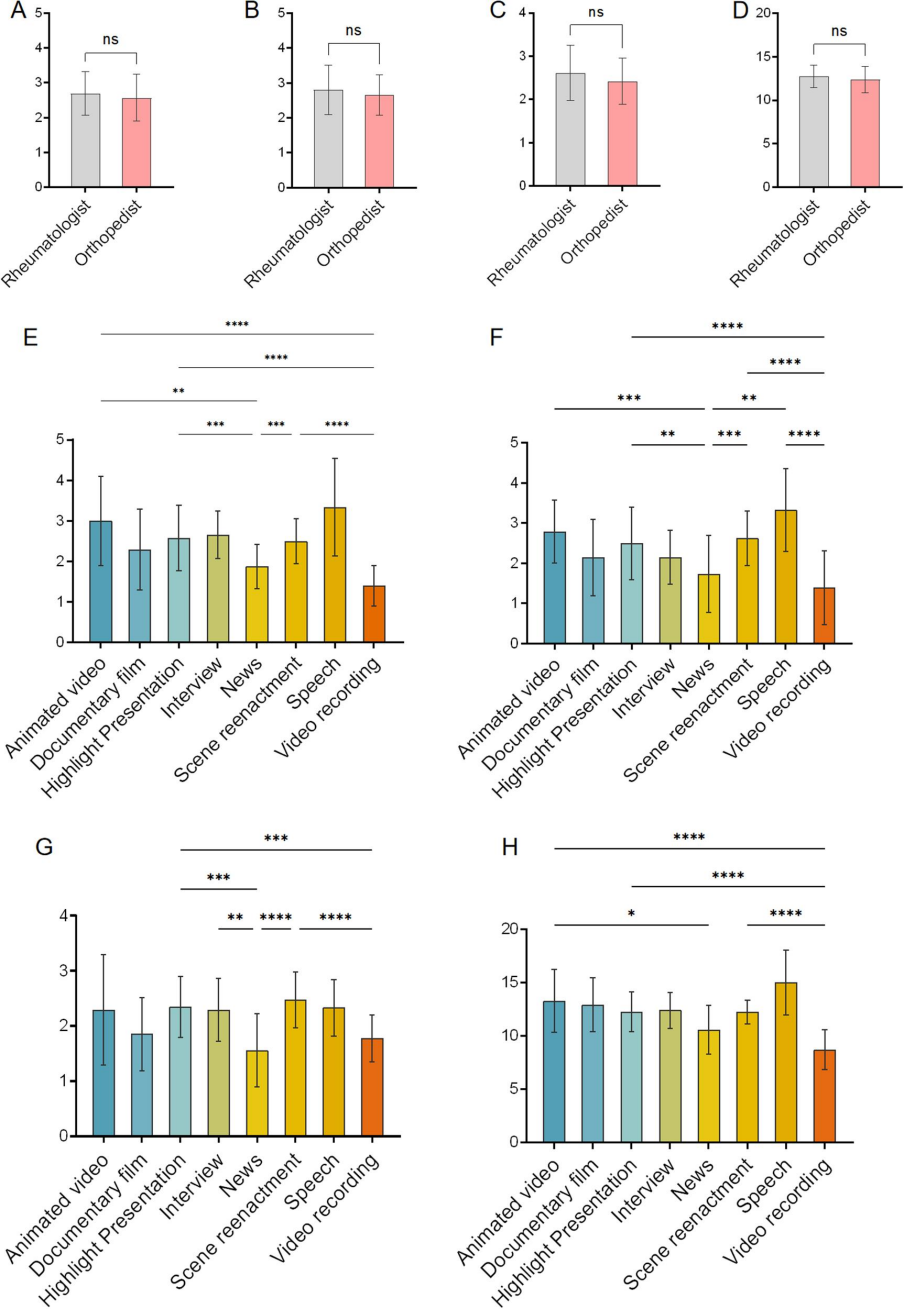
The global quality scale (GQS) score, modified DISCERN score, journal of American medical association (JAMA) score, and video information quality Index (VIQI) score on ankylosing spondylitis from professional subgroup and presentation format. **(A–D)** Comparison of videos uploaded by rheumatologists vs. orthopedists: **(A)** the GQS score, **(B)** the modified DISCERN score, **(C)** the JAMA score, **(D)** the VIQI score; **(E–H)** Comparison of video presentation forms by animated video, highlight presentation, interview, video recording, scene reenactment, speech, and news. **(E)** the GQS score, **(F)** the modified DISCERN score, **(G)** the JAMA score, **(H)** the VIQI score, *P < .05, **P < .01, ***P < .001, ****P < 0.0001.

Videos uploaded by individual users received significantly lower GQS, mDISCERN, and VIQI scores compared with those uploaded by professional physicians and institutions (all *P* < .05; Figures 2A,B,D). Videos from institutions had lower JAMA scores than those from professional physicians (*P* < .001; Figure 2C). Among professional physicians, videos uploaded by rheumatologists and orthopedists showed no significant differences in GQS, mDISCERN, JAMA, or VIQI scores (Figures 3A–D). Regarding video presentation form, animated video achieved the highest GQS and VIQI scores and significantly outperformed news (GQS: mean difference = 1.13, *P* = .002; VIQI: mean difference = 2.726, *P* = .024) and video recording (GQS: mean difference = 1.611, *P* < .001; VIQI: mean difference = 4.569, *P* < .001) (Figures 3E,H). Highlight presentation and scene reenactment also exhibited consistently high quality, with significantly higher GQS and mDISCERN scores than news and video recording (all *P* < .01; Figures 3E,F). In contrast, video recording received the lowest scores across all four assessment tools, performing significantly worse than animated video, highlight presentation, interview, and scene reenactment (all *P* < .001; Figures 3E–G). News videos also demonstrated deficits in reliability (mDISCERN) and information quality (JAMA), with lower scores than animated video, highlight presentation, and scene reenactment (all *P* < .01; Figures 3F,G). Notably, speech videos achieved the highest mDISCERN scores, significantly outperforming news and video recording (all *P* < .001; Figure 3F).

#### 3.3.2 Engagement metrics

Engagement metrics—likes, comments, collections, shares, and fans of video uploaders—varied significantly by uploader type (Table 4). Institutions demonstrated the highest engagement across all metrics except comments, significantly outperforming individual users and professional physicians (all *P* < .001). Among professional physicians, orthopedists received significantly more likes, collections, comments, and fans of video uploaders than rheumatologists (all *P* < .001). Regarding video presentation form, videos presented as interview were most likely to receive higher likes, collections, shares, and fans of video uploaders (all *P* < .001).

**Table 4:** The popularity of videos from different sources and different presentation forms.

Variables	Likes,median (IQR)	Collections,median (IQR)	Comments,median (IQR)	Shares,median (IQR)	Follower count of video uploaders,median (IQR)
Video source					
Individual users	292.00 (4.00, 1,377.00)	81.00 (3.00, 443.00)	34.00 (1.00, 400.00)	29.00 (1.00, 234.00)	627.00 (83.00, 5,213.00)
Institutions	1,744.00 (287.00, 14,799.00)	390.00 (106.00, 2,149.50)	103.50 (19.75, 612.00)	428.00 (62.50, 3,455.00)	296,000.00 (104,000.00, 2,945,250.00)
Non-Professional physicians	564.00 (90.50, 2,219.50)	303.00 (23.00, 741.50)	53.00 (6.00, 279.50)	101.00 (10.50, 942.50)	30,000.00 (8,796.00, 101,000.00)
Professional physicians	121.00 (22.00, 1,131.00)	42.00 (8.25, 322.50)	18.50 (2.00, 130.50)	31.50 (3.00, 261.25)	29,000.00 (5,906.75, 90,000.00)
*P* value	<.001	<.001	0.008	<.001	<.001
Different medical specialties					
Rheumatologist	29.00 (5.00, 198.00)	15.00 (2.00, 98.00)	3.00 (0.00, 40.00)	6.00 (1.00, 61.00)	14,000.00 (3,466.00, 20,000.00)
Orthopedist	268.00 (40.00, 2,060.00)	63.00 (19.00, 512.00)	47.00 (5.00, 201.00)	48.00 (6.00, 700.00)	38,000.00 (9,849.00, 113,000.00)
*P* value	<.001	<.001	<.001	0.002	<.001
Video presentation form					
Highlight presentation	205.00 (22.00, 1,153.50)	65.00 (7.25, 545.75)	19.50 (2.00, 242.25)	44.50 (4.00, 482.50)	15,000.00 (1,726.25, 41,750.00)
Interview	6,585.50 (1,999.00, 23,316.25)	1,166.50 (424.50, 3,522.25)	593.00 (231.75, 927.50)	1,495.50 (577.25, 6,026.00)	1,122,000.00 (80,000.00, 5,255,000.00)
Video recording	312.50 (8.25, 1,211.50)	131.50 (3.00, 427.75)	25.50 (6.00, 381.50)	52.00 (1.00, 185.25)	1,479.50 (186.50, 13,146.25)
Scene reenactment	87.00 (17.00, 1,516.00)	42.00 (9.00, 318.00)	19.00 (1.00, 149.00)	22.00 (2.00, 168.00)	38,000.00 (9,849.00, 113,000.00)
Animated video	295.00 (26.50, 1,655.00)	169.00 (17.00, 726.25)	47.00 (2.50, 186.00)	88.00 (7.25, 1,150.50)	11,660.50 (1,921.50, 90,000.00)
Speech	22.50 (14.00, 36.25)	52.00 (36.00, 90.50)	4.00 (1.75, 4.75)	17.00 (5.25, 35.50)	3,809.00 (717.25, 10,946.25)
News	1,222.00 (95.00, 37,953.00)	139.00 (16.50, 2,038.00)	85.00 (4.00, 612.50)	216.00 (12.00, 4,747.50)	252,000.00 (8,726.00, 3,608,000.00)
Documentary film	1,141.00 (599.25, 2,105.50)	96.50 (37.00, 207.25)	32.00 (10.50, 87.50)	54.50 (14.00, 198.00)	113,000.00 (30,849.50, 152,000.00)
*P* value	<.001	<.001	0.002	<.001	<.001

### 3.4 Correlation analysis

Spearman correlation analysis revealed positive correlations among likes, comments, collections, shares, and fans of video uploaders (all *P* < .001). Duration was not significantly correlated with likes, comments, collections, or shares (all *P* > .05; Table 5). Both collections and duration were positively correlated with GQS, mDISCERN, and VIQI scores (all *P* < .001). Likes were positively correlated with VIQI (*P* < .001), and shares were positively correlated with GQS and VIQI (*P* < .001). No significant correlations were observed between comments or fans of video uploaders and any of the video quality measures (Table 6).

**Table 5:** The correlation analysis between the video variables.

Variables	Likes	Collections	Comments	Shares	Duration (seconds)	Follower count of video uploaders
Likes						
*ρ*	1	0.820	0.728	0.783	0.066	0.231
*P* value	—a	<.001	<.001	<.001	.186	<.001
Collections						
ρ	0.820	1	0.692	0.829	0.067	0.219
*P* value	<.001	—a	<.001	<.001	.176	<.001
Comments						
ρ	0.728	0.692	1	0.677	0.028	0.205
*P* value	<.001	<.001	—	<.001	.532	<.001
Shares						
ρ	0.783	0.829	0.677	1	0.067	0.214
*P* value	<.001	<.001	<.001	—	.176	<.001
Duration (seconds)						
ρ	0.066	0.067	0.028	0.067	1	−0.019
*P* value	.186	.176	.532	.176	—	.685
Follower counts of video uploaders						
ρ	0.231	0.219	0.205	0.214	−0.019	1
*P* value	<.001	<.001	<.001	<.001	.685	—

— Not applicable.

**Table 6:** The correlation analysis between video variables and the video quality.

Variables	GQS	mDISCERN	JAMA	VIQI
Likes				
ρ	0.144	0.134	0.122	0.171
*P* value	0.002	0.004	0.008	<.001
Collections				
ρ	0.203	0.173	0.149	0.236
*P* value	<.001	<.001	0.001	<.001
Comments				
ρ	0.087	0.096	0.099	0.115
*P* value	0.063	0.034	0.028	0.013
Shares				
ρ	0.171	0.152	0.133	0.21
*P* value	<.001	0.001	0.004	<.001
Duration (seconds)				
ρ	0.232	0.205	0.079	0.256
*P* value	<.001	<.001	0.1	<.001
Follower counts of video uploaders				
ρ	−0.045	−0.032	−0.014	−0.028
*P* value	0.324	0.486	0.756	0.531

To further account for potential confounding effects, a multivariable linear regression analysis was performed using the platform-wise standardized collection score as the dependent variable. After adjustment for platform, days since upload, follower count, and video duration, higher mDISCERN scores were independently associated with lower relative collection levels (*P* < .01), while days since upload and follower count remained significant positive predictors.

## 4 Discussion

### 4.1 Principal findings

This study provides the first systematic comparison of the information quality and reliability of ankylosing spondylitis–related short videos across TikTok, Bilibili, and rednote. Overall, the analyzed videos demonstrated a moderate level of information quality, consistent with prior studies of disease-related content on short-video platforms, which have similarly reported substantial heterogeneity and generally modest median quality levels (27–31). Across platforms, uploader identity and presentation format emerged as key factors associated with video quality. Videos produced by physicians consistently achieved higher scores across GQS, mDISCERN, JAMA, and VIQI, while more structured formats—such as animated videos and lecture-style explanations—were associated with superior quality and reliability. In contrast, casually recorded or experience-based videos tended to perform less well across multiple evaluation dimensions. With respect to user engagement, our findings indicate a nuanced and metric-dependent relationship between information quality and engagement behaviors. In unadjusted analyses, collections were positively correlated with video quality, whereas no consistent associations were observed with likes, comments, or shares. After accounting for platform characteristics and exposure-related factors, information quality was not an independent driver of relative engagement, and mDISCERN was inversely associated with standardized collection levels. Together, these findings suggest that while information quality may influence certain forms of user interaction, it does not necessarily translate into broader visibility or popularity on short-video platforms, a pattern that has been observed across multiple health communication studies in social media contexts (32–34).

### 4.2 Factors associated with video popularity

Videos related to ankylosing spondylitis received up to 200,000 likes, with some videos exceeding 20,000 saves and shares, indicating strong public interest in this condition. In China, this heightened attention may be partly attributable to several well-known actors and singers publicly disclosing their diagnoses, which increased public awareness, as celebrity involvement has been shown to influence the popularity of information on social media platforms (35). Consistent with this explanation, multiple videos in our sample used disease narratives involving specific celebrities as introductory hooks to attract viewers. User engagement metrics—including likes, comments, collections, and shares—have been reported to partially reflect audience preferences and video popularity (36, 37). Our study found that TikTok outperformed Bilibili and rednote across all engagement indicators (*P* < 0.01), whereas videos on Bilibili were significantly longer in duration than those on TikTok or rednote. However, because engagement metrics are influenced by platform accessibility, cross-platform comparisons should be interpreted as reflecting differences in platform ecosystems rather than intrinsic differences in content appeal. This interpretation is consistent with previous TikTok-related research showing that platform design and trend dynamics strongly shape interaction behaviors (38). TikTok's algorithm is optimized for rapid consumption of short-form, high-frequency content that facilitates quick interaction, whereas Bilibili tends to favor long-form, knowledge-dense videos that require sustained viewer attention and may therefore generate lower immediate engagement (30, 39).

Our study further found that videos uploaded by institutional accounts demonstrated significantly higher engagement across all metrics except comments. This pattern may be attributable to the higher brand credibility, consistent content output, and greater promotional resources typically associated with institutional accounts, all of which can enhance audience trust and willingness to share, thereby increasing likes, collections, and reposts (40, 41). In addition, videos produced by orthopedic specialists achieved higher engagement than those created by rheumatologists. This difference may stem from broader public familiarity with musculoskeletal symptoms, a larger patient population affected by orthopedic conditions, and the inherently visual characteristics of orthopedic content—such as posture correction or exercise demonstrations—which may reduce cognitive barriers and preferentially increase likes and saves (42, 43). Moreover, videos presented in an interview-style format—particularly those featuring physicians or patients—were more likely to receive higher numbers of likes, collections, shares, and follower growth. This trend may reflect the perceived authenticity and enhanced emotional resonance of such content (32).

### 4.3 Factors associated with video quality

Our study found that uploader identity exerted a significant influence on information quality. Videos uploaded by physicians achieved higher scores across multiple quality assessment instruments and clearly outperformed those uploaded by individual users or institutional accounts. This may reflect physicians' deeper professional knowledge base and their greater adherence to evidence-based information and clinical ethical standards during content creation. Similar findings have been consistently reported in studies of disease-related videos, such as analyses of pregnancy-related rheumatoid arthritis and umbilical hernia videos on YouTube, in which physician-produced content consistently achieved higher GQS and mDISCERN/DISCERN scores than videos created by non-professionals or patients (44, 45), as well as in evaluations of cardiac rehabilitation videos on YouTube (46), which also used GQS, JAMA, and mDISCERN and found predominance of low-quality content and associations between professional sources and higher scores. Taken together, these convergent findings underscore the importance of professional medical expertise in health information videos. However, higher information quality does not necessarily translate into higher user engagement, a pattern that has been reported in multiple disease-related video studies (47–49). The findings of the present study further support this observation, although the underlying mechanisms require integrated interpretation in relation to specific engagement behaviors and platform contexts (see Section 4.4).

Video presentation format was also closely associated with information quality. In this study, animated and lecture-style videos received the highest quality scores, likely due to clearer structural organization, scripted delivery, and more effective visual guidance (50, 51). Similarly, Ni et al. (52) reported that videos featuring physician-led explanations or question-and-answer formats significantly outperformed patient experience–sharing videos on TikTok in terms of mDISCERN, GQS, JAMA, and VIQI scores. In contrast, casually recorded videos lacked organizational structure and visual guidance and performed poorly across multiple evaluation dimensions. This observation is consistent with prior analyses of pregnancy-related rheumatoid arthritis and sudden sensorineural hearing loss videos, suggesting that content production style itself is a critical determinant of information quality. With respect to platform differences, videos on Bilibili demonstrated better reliability as measured by mDISCERN compared with those on TikTok and rednote. This may be related to Bilibili's community culture, which tends to favor longer-duration, knowledge-dense content, as well as the relatively high proportion of “professional lectures or courses” on this platform (53, 54). However, no significant differences were observed among the three platforms in overall quality perception (GQS), information quality (VIQI), or authority indicators (JAMA). Similar findings were reported in an analysis of stroke-related videos (55), which showed comparable quality scores across platforms, suggesting that platform choice alone does not guarantee higher information quality. In line with this, a previous study on radiotherapy-related short videos also noted that platform characteristics have limited influence on video quality and that platform selection alone cannot ensure information accuracy (56). In addition, our content analysis revealed a pronounced imbalance in topic coverage. Ankylosing spondylitis–related videos predominantly focused on clinical manifestations and treatment, whereas epidemiology, etiology, and prognosis were markedly underrepresented. Although short-video time constraints may partly limit content completeness, given that long-term exercise adherence, posture management, and continuous pharmacotherapy are core determinants of AS outcomes (57), the relative lack of rehabilitation- and prognosis-related content more likely reflects a disease-specific gap in public health communication. This knowledge deficit may limit patients' understanding of long-term disease management priorities and negatively affect self-management and functional outcomes.

### 4.4 User engagement behaviors and video quality: correlation analysis

In this study, unadjusted correlation analyses showed that video collections were positively associated with multiple information quality scores, suggesting that users may, to some extent, prefer to retain content they perceive as more informative. However, after platform-wise standardization and simultaneous adjustment for platform differences, video upload time, creator popularity, and video duration, information quality was no longer an independent determinant of user engagement. This finding indicates that user engagement behaviors on short-video platforms are shaped by multiple interacting factors rather than information quality alone. These results further suggest that the relationship between information quality and user engagement is highly context-dependent and metric-dependent. For retention-oriented engagement behaviors such as collections, information quality may still play a role; in contrast, for more immediate and emotion-driven interactions—such as likes, comments, and shares—its influence appears limited. Importantly, this complex relationship is not unique to ankylosing spondylitis–related content. Previous studies on sudden sensorineural hearing loss have similarly reported weak associations between audience engagement and video quality, with some studies even identifying negative correlations between these measures (58). Moreover, in a multi-platform analysis of stroke-related videos, Nie et al. (55) found no significant association between basic video characteristics and information quality, and their machine learning models were unable to accurately predict video quality. Collectively, these cross-disease findings indicate that user engagement metrics are unreliable proxies for assessing the credibility of health information videos. Overall, our findings further emphasize that, within algorithm-driven short-video environments, the relationship between user engagement and information quality should be interpreted with caution. Reliance on interaction metrics alone to evaluate the credibility of health information may lead to systematic underestimation of high-quality content.

### 4.5 Recommendations based on study findings

First, for healthcare professionals, greater involvement of physicians in the creation of short-video health education content should be encouraged. Based on our findings, adopting more structured and engaging presentation formats—such as animations, scenario-based interviews, and lecture-style explanations—may help balance informational accuracy with audience accessibility, thereby improving the overall quality of medical content disseminated on short-video platforms. Second, for platform administrators, it may be valuable to explore the integration of content quality indicators into recommendation or moderation frameworks, rather than relying exclusively on user engagement metrics for content ranking. Given the complexity of platform algorithms and attention dynamics, such measures should be considered as part of a broader strategy aimed at improving the visibility of high-quality health information. In parallel, enhancing certification and verification systems for health content creators may further assist users in identifying professionally credible sources. Third, for policymakers and professional associations, promoting the development of standardized guidelines for short-video–based medical education represents a potential long-term direction. Such initiatives could include the establishment of authoritative, structured, and disease-specific medical video repositories to support consistent and reliable health communication across platforms. Finally, for the general public, strengthening digital health literacy remains essential. Users should be aware that engagement metrics—such as likes, comments, and shares—do not necessarily reflect the accuracy or reliability of medical information. Prioritizing content produced by certified and professionally qualified healthcare providers may help reduce the risk of misinformation exposure.

### 4.6 Study significance and contributions

This study provides the first systematic evaluation of ankylosing spondylitis–related short videos across TikTok, Bilibili, and rednote, thereby addressing an important evidence gap in the understanding of disease-related health communication on Chinese social media platforms. By applying multiple validated assessment instruments, this research demonstrates that uploader identity and presentation format are consistently associated with information quality, with physician-produced and structurally organized videos achieving higher quality and reliability scores. Beyond descriptive quality assessment, this study contributes to the existing literature by clarifying the nuanced relationship between information quality and user engagement in short-video environments. Our findings suggest that while certain engagement behaviors, such as content saving, may reflect users' perceived informational value, overall engagement metrics are not independently driven by information quality after accounting for platform characteristics and exposure-related factors. These results underscore the limitations of relying solely on engagement indicators to infer the credibility of health information. Collectively, this study advances current understanding of the short-video health information ecosystem by highlighting both the determinants of high-quality content and the structural challenges faced by professional medical information in algorithm-driven attention economies. The findings offer empirically grounded insights that may inform future research, platform-level governance discussions, and public health communication strategies in rapidly evolving digital media contexts.

### 4.7 Limitations

Several limitations of this study should be acknowledged. First, the cross-sectional design limits the ability to capture temporal changes in video quality and user engagement, and precludes assessment of how evolving platform algorithms may influence content visibility and dissemination over time. Although upload age was accounted for analytically, the absence of longitudinal sampling prevents evaluation of dynamic trends or event-driven fluctuations. Second, while the sample size met statistical requirements and was balanced across platforms, it represents only a small fraction of the vast volume of ankylosing spondylitis–related content available on short-video platforms. In addition, all videos were collected within a predefined and relatively short time window, which may have been influenced by contemporaneous events such as public awareness campaigns, media coverage, or celebrity disclosures. These time- and event-specific effects were not explicitly modeled and may have affected content themes and engagement patterns. Third, this study employed fixed Chinese disease-specific keywords for video retrieval. Although this strategy enhanced search standardization and reproducibility, it may have excluded relevant videos using dialectal expressions, synonymous terms, or metaphorical language. Future studies incorporating broader lexical strategies or platform-specific hashtag analyses may improve content coverage. Fourth, although overtly promotional videos lacking substantive educational value were excluded during screening, we did not perform a dedicated coding of commercial intent, such as advertisements, paid courses, or clinic promotion. The potential influence of commercial motivation on information quality and user engagement therefore could not be systematically evaluated and warrants further investigation. Fifth, despite the use of multiple widely adopted and validated assessment instruments, quality scoring inherently involves a degree of subjectivity. While inter-rater reliability was formally assessed, future research may benefit from integrating automated or semi-automated evaluation approaches, such as machine learning–based video content analysis, to enhance objectivity and scalability in the Chinese short-video context. Finally, although the use of newly registered accounts helped reduce personalization bias, this approach cannot fully replicate the long-term recommendation environment experienced by real users. Future studies employing longitudinal designs, simulated user profiles, or experimental exposure models may provide deeper insight into algorithmic influences on health information dissemination.

## 5 Conclusion

Ankylosing spondylitis–related short videos on TikTok, Bilibili, and rednote are predominantly uploaded by physicians and are often presented in relatively structured formats, such as animations or lecture-style explanations. Despite this, the overall quality and reliability of available content remain moderate. Although unadjusted analyses suggested that content saving may reflect perceived informational value, information quality was not a consistent independent driver of relative engagement after adjustment for platform characteristics and exposure-related factors. These findings suggest that user engagement metrics alone may not reliably reflect the credibility or educational value of health information on short-video platforms. From a public health perspective, viewers should prioritize content produced by verified medical professionals, and platform stakeholders and healthcare providers should consider strategies that enhance both the quality and visibility of accurate health information, rather than relying solely on engagement-driven dissemination mechanisms.

## Data Availability

The original contributions presented in the study are included in the article/Supplementary Material, further inquiries can be directed to the corresponding author.
