# Supplementary material for: Short-form video platforms as a source of ankylosing spondylitis information: a cross-sectional content analysis
Source: Front Digit Health. 2026 Mar 4;8:1757584. doi: 10.3389/fdgth.2026.1757584 (PMC12996211; doi:10.3389/fdgth.2026.1757584)
Supplement: Supplementary file 1 [file Datasheet1.docx]

**Supplementary Materials**

**Supplementary Table 1. The Global Quality Score (GQS) quality criteria.**

| **Item features** | **Points** |
| --- | --- |
| Poor quality; poor flow of the videos; most information missing; not at all useful for patients | 1 |
| Generally poor quality; some information listed, but many important topics missing; of very limited use to patients | 2 |
| Moderate quality; suboptimal flow; some important adequately discussed, but other information poorly discussed; somewhat useful for patients | 3 |
| Good quality and generally good flow; most of the relevant information listed, but some topics not covered; useful for patients | 4 |
| Excellent quality and flow; very useful for patients | 5 |

**Supplementary Table 2.** **The Modified DISCERN (mDISCERN) quality criteria.**

| **Reliability Score** |
| --- |
| 1. Is the video clear, concise, and understandable? |
| 2. Are valid sources cited? |
| 3. Is the content presented balanced and unbiased? |
| 4. Are additional sources of content listed for patient reference? |
| 5. Are areas of uncertainty mentioned? |

* (1 point for answer ‘yes’, 0 point for answer ‘no’)

**Supplementary Table 3**. **The Journal of the American Medical Association (JAMA) benchmark criteria.**

| Score* | Score component | |
| --- | --- | --- |
| 1 score | Authorship | Author and contributor credentials and their affiliations should be provided. |
| 1 score | Attribution | Clearly lists all copyright information and states references and sources for content. |
| 1 score | Currency | Initial date of posted content and subsequent updates to content should be provided. |
| 1 score | Disclosure | Conflicts of interest, funding, sponsorship, advertising, support, and video ownership  should be fully disclosed. |

*The criteria of each aspect were scored separately, and 1 point for each criterion with a total score of 4 points.

**Supplementary Table 4.** **The** **VIQI (Video Quality Instrument) quality criteria.**

| Dimension | Score component | |
| --- | --- | --- |
| VIQI-1 | Information Flow | How well is the information presented in a structured, sequential, and easy-to-follow manner? 1-5 |
| VIQI-2 | Information Accuracy | To what extent is the medical information provided correct, complete, and up-to-date? 1-5 |
| VIQI-3 | Video Quality | This parameter is the sum of 5 binary sub-items. Each present sub-item scores 1 point, absent scores 0 points. The total for this parameter is the sum, ranging from 0 to 5.)  • Use of still images(0 or 1)  • Use of animation (0 or 1)  • Inclusion of community interviews 0 or 1)  • Presence of video subtitles (0 or 1)  • Inclusion of a report summary (0 or 1) |
| VIQI-4 | Precision | How well does the video content align with and deliver on the promise of its title? 1-5 |

Total VIQI Score (Sum of Scores for Parameters 1, 2, 3 (subtotal), and 4) 4-20

**Supplementary Table 5. Inter-rater agreement for content completeness across seven medical domains**

| Domain | Cohen’s κ (95% CI) |
| --- | --- |
| Epidemiology | 0.898 (0.824–0.972) |
| Etiology | 0.887 (0.810–0.964) |
| Clinical manifestation | 0.920 (0.873–0.966) |
| Diagnosis | 0.939 (0.894–0.983) |
| Treatment | 0.920 (0.875–0.964) |
| Prevention | 0.893 (0.800–0.986) |
| Prognosis | 0.914 (0.839–0.989) |

Table note：

Cohen’s kappa coefficients were calculated to assess inter-rater agreement for binary content completeness items (presence vs. absence).
